# Polypharmacy in Pediatric Palliative Care: Exploring Discrepancies Between Physicians and Pharmacists

**DOI:** 10.3390/children12020124

**Published:** 2025-01-24

**Authors:** Daniele Mengato, Anna Zanin, Fernando Baratiri, Lisa Pivato, Laura Camuffo, Franca Benini, Francesca Venturini

**Affiliations:** 1 Hospital Pharmacy Department, Azienda Ospedale-Università Padova, Via Giustiniani, 35128 Padua, Italy; lisa.pivato@aulss1.veneto.it (L.P.); laura.camuffo@aopd.veneto.it (L.C.); francesca.venturini@aopd.veneto.it (F.V.); 2 Palliative Care and Pain Service, Department of Women’s and Children’s Health, University of Padua, Via Giustiniani, 3-35128 Padua, Italy; anna.zanin@aopd.veneto.it (A.Z.); fernando.baratiri@aulss3.veneto.it (F.B.); franca.benini@aopd.veneto.it (F.B.)

**Keywords:** off-label, palliative care, polypharmacy, pharmacovigilance, regulatory compliance, clinical effectiveness, clinical pharmacy, pediatrics

## Abstract

**Background/Objectives**: Off-label drug use is prevalent in pediatric care, particularly in pediatric palliative care (PPC), due to the scarcity of pediatric-specific formulations and clinical trials. Differences in perception between healthcare professionals regarding off-label prescriptions underscore the complexity of this practice and highlight the need for improved collaboration to optimize therapeutic outcomes. **Methods**: A cross-sectional observational study was conducted from August to October 2021 at the PPC center of the University Hospital of Padova, Italy. Data were collected from medical records of 169 patients. Off-label prescriptions were independently assessed by two physicians and two clinical pharmacists using respective reference sources. Discrepancies were resolved through consensus. Statistical analyses included the χ^2^-test for categorical variables and *t*-tests for continuous data. **Results**: Among the 993 drug prescriptions analyzed, the pharmacists reported a higher proportion of off-label uses (32.9%) compared to the physicians (18.4%; *p* < 0.05). After a consensus, 26.5% of the prescriptions were identified as off-label, with 67.9% due to indications, 49.6% due to dosage, and 44.4% due to age discrepancies. **Conclusions**: This study suggests a high prevalence of off-label prescribing in pediatric palliative care (PPC) and highlights differing professional perspectives, underscoring the potential benefits of exploring standardized protocols and enhanced interdisciplinary collaboration. Enhanced communication between healthcare providers, alongside the development of registries and clinical trials, is essential for improving the safety and efficacy of off-label drug use in pediatric populations. A flexible regulatory framework and customized galenic formulations could further support these goals.

## 1. Introduction

Children, defined as individuals aged 0 to 18 years, require targeted research to investigate the pharmacological and toxicological aspects of medications, ensuring the development of safe, effective, and high-quality drugs [1]. Although clinical trials involving pediatric populations are essential for the safe use of medications in this demographic, they often encounter various ethical challenges and concerns. Consequently, many medications administered in pediatric care are prescribed off-label—meaning they are used outside the authorized indications specified in the Summary of Product Characteristics (SmPC)—resulting in inadequate documentation regarding dosing, efficacy, and safety [2]. A single prescription may be classified as off-label for various reasons, the most common of which are summarized in Table 1 [3].

Off-label drug use is a pervasive practice in pediatric prescribing, particularly within hospital settings. Recent studies emphasize that young children, especially infants and toddlers, are the most frequent recipients of undocumented prescriptions [3,4]. In neonatal and pediatric hospital care, the prevalence of off-label drug use exhibits significant variability, ranging from 10% to 65%, depending on the clinical context. In outpatient pediatric settings, this range is slightly narrower, reported between 11% and 31% [3,4,5,6]. Among therapeutic categories, analgesics and antibiotics are most commonly prescribed off-label in hospitals [3,5,6]. Frequently used off-label medications in these settings include morphine, salbutamol, heparin, and various cardiovascular agents [5,6,7].

Assessing whether a treatment constitutes off-label use presents multifaceted challenges spanning clinical, ethical, and communication domains. Clinically, the absence of robust scientific evidence and an increased risk of adverse drug events (ADEs) are major concerns, as many off-label indications lack support from high-quality clinical trials. Despite regulatory efforts to incentivize pediatric drug research, off-label prescribing remains widespread due to the limited availability of pediatric-specific formulations and the paucity of age-appropriate clinical trials [8,9]. The lack of standardized protocols further complicates monitoring for treatment efficacy and safety. Additionally, the absence of comprehensive registries for off-label use impedes large-scale data collection, restricting the ability to evaluate such treatments systematically.

The vulnerability of infants and children to off-label drug use is compounded by their immature hepatic and renal functions and their distinct pharmacokinetic and pharmacodynamic profiles, which require careful dose adjustments. This underscores the urgency of generating more evidence on the safety and efficacy of off-label medications. Critically ill neonates and infants, among the most fragile pediatric populations, experience the highest levels of undocumented drug use, reinforcing the need for targeted research in this area [10]. Existing studies suggest that ADEs associated with off-label use often arise from incorrect dosing or inappropriate routes of administration rather than the off-label indication itself [11].

Pediatric palliative care (PPC) globally focuses on addressing the physical and psychological needs of children with life-limiting or life-threatening conditions, as well as the needs of their families [12]. Recent estimates suggest that approximately 10,600 children in Italy require specialized PPC services [13]. However, the nationwide PalliPed project revealed that fewer than two out of every ten children have access to these services, indicating a significant need for improvements in service delivery and a more efficient allocation of resources [14]. These children often present many comorbidities and are exposed to polypharmacy, where the concurrent use of multiple medications increases the likelihood of drug–drug interactions, cumulative toxicities, and medication errors [15]. This is the reason why we selected the PPC context to analyze off-label prescription, as it exemplifies this complexity, since the need to manage multiple comorbidities frequently necessitates polypharmacy, further heightening these risks [15,16].

The differing perceptions of off-label use between clinical pharmacists and physicians are crucial in understanding the broader implications for patient care. Healthcare professionals, particularly pharmacists and physicians, may have differing perceptions of off-label prescribing, with pharmacists tending to emphasize legal and ethical considerations, while physicians focus on clinical efficacy and patient outcomes. Pharmacists advocate for rigorous documentation and monitoring practices compared to physicians, who tend to prioritize clinical efficacy and patient-centered outcomes [17,18]. This divergence in perspectives highlights the critical need for enhanced interdisciplinary communication and collaboration to optimize the safety and effectiveness of off-label prescribing, particularly for vulnerable pediatric populations [19,20]. Such teamwork can bridge gaps in knowledge and ensure a comprehensive approach to optimizing the safety and effectiveness of off-label prescribing—particularly in pediatric populations, who are more vulnerable to the risks associated with such practices.

This study aims to explore the perspectives of physicians and clinical pharmacists on off-label prescribing within PPC, focusing on whether their views diverge and identifying potential gaps in knowledge regarding pediatric treatments. Given that PPC is notably susceptible to off-label prescriptions and that many patients experience polypharmacy, this context provides a compelling framework for this research proposal.

## 2. Materials and Methods

### 2.1. Study Design

A cross-sectional observational study was carried out between August and October 2021 in the PPC center of the University Hospital of Padova, Italy. Data were initially collected from medical records, followed by a separate analysis of off-label treatments by two groups: two physicians from the PPC center who were not the main prescriber and two clinical pharmacists from the Hospital Pharmacy Unit. Each group assessed the type of prescription (on-label or off-label) and its rationale, using their respective reference sources: the pharmacists consulted the SmPC and guidelines from the Italian Medicines Agency (AIFA), while the physicians relied on the SmPC, Clinical Decision Support Softwares (CDSSs), and international guidelines. Discrepancies within each group were resolved by a multidisciplinary team, and the final assessment was based on a common consensus.

### 2.2. Inclusion Criteria

The study included patients having at least one access at the PPC center and with at least one prescribed drug treatment and an age of less than 23 years. The data collected included age, gender, primary diagnosis, number and type of medications, frequency and route of administration, and ability to self-administer. The drugs were categorized by Anatomical Therapeutic Chemical (ATC) classification for further analysis.

### 2.3. Data Collection

Clinical data, such as medical characteristics, were obtained from medical records and pharmacological reviews of patients’ therapies during the study period. For new prescriptions, further analysis was conducted. The physicians and pharmacists independently evaluated whether each treatment was on-label or off-label, based on the patient’s condition and the drug’s authorized indication. Guidelines issued by AIFA were also consulted, particularly those on early drug access, cross-referenced with dosage and SmPC recommendations [21] and cross-checked with the patients’ own dosage and with the sheet patient information leaflets. Considering the complexity of some pediatric pathologies, including rare diseases, it was necessary to identify signs and symptoms of the single pathology by obtaining information from what is present in the bibliography in the Italian register of rare diseases and orphan drugs [22]. The medical approach to the analysis was different: the physicians traced the entire pathological picture of the individual patient to the pre-existing international guidelines, thus considering all the symptoms resulting from the primary diagnosis and the individual situation. It was thus defined whether a treatment was then appropriate to the individual disease situation. At the end of the evaluations, a consensus between the pharmacists and clinicians allowed conflicting assessments to be resolved. This consensus was reached through an online meeting where all patient charts were discussed in detail to review and resolve disagreements in the detection of off-label prescriptions. During the meeting, a systematic analysis of each patient’s diagnosis, treatment duration, and, where applicable, the evolution of their clinical condition was conducted. To ensure a robust and consistent assessment, the evaluation was supported by reference to multiple authoritative sources, including the AIFA guidelines, the SmPC for each drug, and relevant clinical practice guidelines.

Discrepancies in the off-label detection process were addressed by fostering a collaborative discussion among all the participants. Cases with conflicting interpretations were revisited in light of the standardized references and patient-specific details. When disagreement persisted, the majority decision was adopted following a thorough review of the evidence and guidelines. In particularly complex cases, additional literature or expert opinions were sought to achieve consensus. This iterative process ensured the accurate classification of prescriptions, balancing strict adherence to regulatory frameworks with the practical nuances of individualized patient care.

### 2.4. Statistical Analysis

Normally distributed continuous data were reported as mean ± SD and compared using the two-sided Student’s *t*-test. Non-normally distributed continuous data were reported as the median and interquartile-range (IQR) and compared using the Mann–Whitney test. Categorical variables were expressed as frequencies and percentages and were analyzed using the χ^2^-test with Yates’s correction or Fisher’s exact test, whichever was most appropriate.

### 2.5. Ethical Considerations

This study adhered to the Good Clinical Practice (GCP) guidelines and European directives 2001/20/CE and ISO 14155, and in agreement with the local regulations. The final protocol and its amendments were reviewed and approved by the local Ethical Committee (CE) with the number 197n/AO/21. Written informed consent was obtained from a parent and/or legal guardian.

## 3. Results

### 3.1. Baseline Characteristics of the Included Patients

The study included a total of 169 patients, with a near-equal distribution of males (49%) and females (51%), having a median age of 12.5 years (interquartile range [IQR] 6–18). The age distribution revealed that 5.9% were infants (1 month to 2 years), 19.5% were preschool-aged children (3–5 years), 22.5% were school-aged children (6–11 years), and 38.5% were adolescents (12–18 years). Additionally, 13.6% of the population were adults (≥18 years). The mean duration of disease history among the participants was 10.4 years (±6.0), with a median of 11 years (IQR 2–18). The most common primary diagnosis categories included neurologic disorders (41.4%), musculoskeletal conditions (28.4%), and genetic–metabolic disorders (16.0%). Notably, 90.5% of patients had congenital or perinatal onset conditions. To better understand the complexity of each patient’s therapeutic regimen, treatments and supplements were also considered. A total of 993 pharmacological treatments were analyzed, categorized as either chronic therapies or as-needed medications. On average, each patient was taking 5.9 medications, with 52.7% of patients engaged in polypharmacy (defined as the concurrent administration of five or more drugs per day), while 19.5% were identified with excessive polypharmacy (ten or more drugs per day). These patients were taking an average of 13.5 medications daily, with a Medication Regimen Complexity Index (MRCI) score of 44.8 [23]. A substantial 44.4% of the participants faced a medication burden, which was characterized by polypharmacy plus at least two drug administrations per night. Additionally, only 22.5% of the patients were involved in self-administration of their medications. See Table 2 for further details.

These findings underscore the complex medication regimens frequently encountered in pediatric populations, particularly those with chronic conditions, and highlight the need for careful management to mitigate potential risks associated with polypharmacy, including adverse drug reactions and drug–drug interactions.

### 3.2. Off-Label Analysis

**Figure 1:**
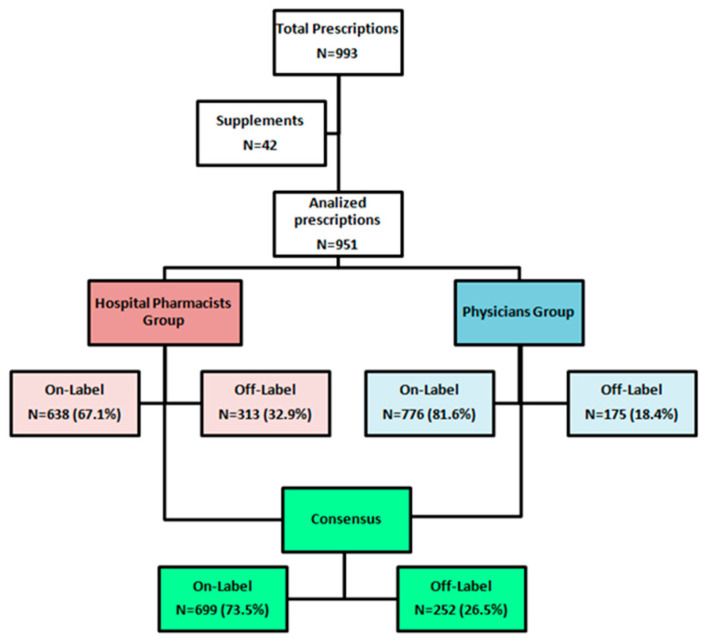
Prescriptions review process.

When focusing on drug prescriptions alone, a comparison of off-label uses revealed that out of 951 total drug treatments, the clinicians identified 175 (18.4%) instances of off-label use, while the pharmacists documented 313 off-label uses, representing 32.9% of all the prescriptions reviewed (*p* < 0.05). For a detailed overview of the review process, please refer to Figure 1.

For each identified off-label use, a detailed analysis was conducted to categorize the specific type of utilization. The analysis evaluated the incidence rates of off-label use across various dimensions, including clinical indication, patient age, route of administration, dosage regimen, and other relevant factors. Differences were notable in classifications such as use for different indications (25.9% identified by the pharmacists vs. 57.1% identified by the clinicians, *p* < 0.05), route of administration (9.9% vs. 4.6%, *p* = 0.04), and dosage (44.1% vs. 18.3%, *p* < 0.05). However, no significant differences were observed for age-related off-label uses (*p* = 0.27). Moreover, the physicians more frequently reported “other” non-recommended uses (17.1% vs. 0.9%, *p* < 0.05), which included unauthorized manipulations. These findings highlight the varying perspectives and rigor applied by the two groups. Detailed findings are presented in Table 3.

Among the drugs most frequently classified as off-label in the physicians’ group were azithromycin, salbutamol, and cannabinoids. In contrast, the pharmacists’ group identified cholecalciferol, lansoprazole, antibiotics, and macrogol as the most prevalent off-label medications. The pharmacists were more likely to recognize off-label uses by route of administration, in particular related to the administration of drugs via enteral tubes, while the physicians were more likely to detect off-label uses, such as unauthorized manipulations, as they also had access to the individual administration data.

In the consensus process conducted after the evaluations by the clinicians and pharmacists, 252 prescriptions (26.5%) were identified as off-label. The majority of these were categorized as off-label due to indication, representing 171 cases (67.9%). Additionally, 49.6% of the off-label uses were related to dosage deviations, and 44.4% were associated with age discrepancies. Notably, 132 prescriptions (52.4%) were off-label for multiple reasons.

**Figure 2:**
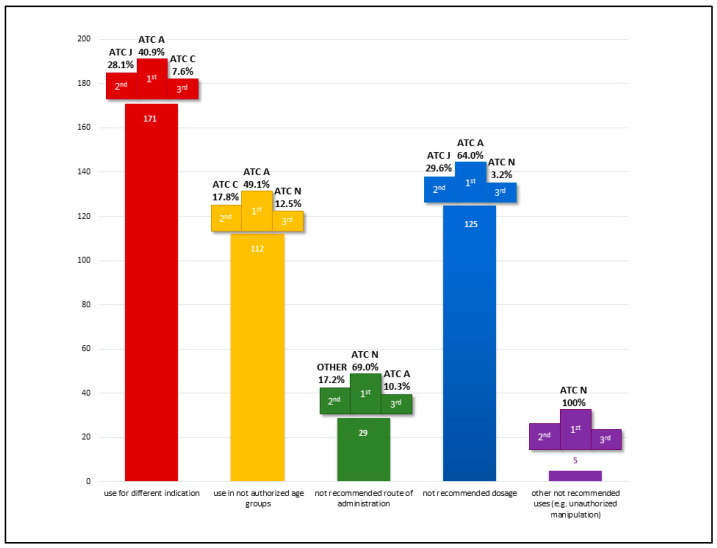
Off-label definition and most common uses per Anatomical Therapeutic Chemical (ATC) classification according to clinicians–pharmacists consensus.

The distribution of off-label uses by the first ATC level is detailed in Figure 2, highlighting the top three most represented therapeutic classes across use categories. Class A (Alimentary Tract and Metabolism) emerged as the most represented category overall, as well as for off-label uses by indication, age, and dosage. This predominance is primarily attributable to the chronic use of proton pump inhibitors and macrogol.

Class J (Anti-infective Drugs for Systemic Use) accounted for approximately one-fifth of the total off-label prescriptions, driven predominantly by their application in long-term prophylaxis among frail patient populations.

Regarding off-label uses by route of administration, Class N (Nervous System) was the most prominent. This reflects the frequent administration of antiepileptic drugs via enteral tubes, a common practice in PPC patients.

### 3.3. Off-Label Prevalence in Polypharmacy Patients

**Figure 3:**
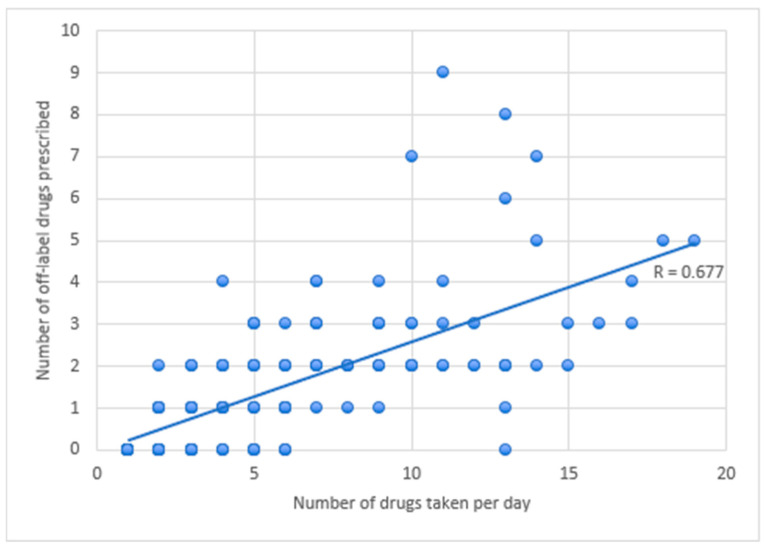
Correlation analysis between the number of drugs taken per day and off-label uses.

Overall, among the 89 patients in the cohort taking five or more drugs daily, the incidence rate of off-label prescriptions was 26.7%, aligning with the results from the consensus. This trend remained consistent even when focusing solely on patients with excessive polypharmacy (≥10 drugs per day), where the off-label prescription rate was 25.9%. Conversely, in patients taking fewer than five drugs per day, the percentage of off-label prescriptions decreased to 22.4%. Notably, 11 patients (12.3% of those with polypharmacy) had no off-label prescriptions; of these, only one was taking ten or more drugs per day. A correlation analysis (Figure 3) revealed a positive relationship between the number of drugs taken and the incidence of off-label prescriptions, with an R value of 0.677, which was statistically significative (*p* < 0.05).

## 4. Discussion

Our analysis highlights the percentage (26.5%) of off-label prescriptions in this PPC cohort and the difference between physicians and pharmacists regarding its perception. This divergence underscores the complexity inherent in off-label prescribing practices and the need for a collaborative framework [19].

This result can highlight both accuracy and strict adherence to regulation and guidelines by the pharmacists on one side and underestimation by the clinical physicians on the other. This is probably because the common and routine use of these drugs in such a subspecialized context, despite their original indication, can lead physicians to become used to their prescription in this way and then to underestimate their off-label prescribing rate [24].

Finding common ground between the structured regulatory approach of hospital and clinical pharmacists and the patient-centered clinical perspective of physicians is critical for guaranteeing medication treatment effectiveness and safety. The different recognition between clinical physicians and pharmacist of off-label drugs for some specific items such as duration of treatment, route of administration, or even the dosage reveal significant variations in their approaches. Physicians have a thorough awareness of their patients’ clinical problems and unique treatment needs. They are continually monitoring symptoms and clinical patient status with regular follow-up after starting a drug to detect the effectiveness of the treatment and also its potential adverse effects, sometimes proposing off-label drugs as the only possibility for an effective treatment. This probably results in their not recognizing as valuable some off-label indications of specific drugs, such as prolonged duration issues, whereas pharmacists are more focused on pharmacological characteristics, drug interactions, and adherence to current regulations. The identification of commonly used off-label medications in this study, such as azithromycin and macrogol, suggests that further investigation into the development of standardized protocols and improved communication between healthcare providers, scientific societies, and pharmacists may be beneficial. Establishing regular interdisciplinary meetings can facilitate ongoing dialogue about off-label drug use, ensuring that all parties are aligned on treatment goals and strategies. This proactive approach is particularly important in pediatric populations, where the complexities of off-label prescribing may be influenced by factors such as polypharmacy and the unique pharmacokinetic profiles of children. In PPC, the issue of polypharmacy is particularly pressing, as more than 50% of children are experiencing polypharmacy, defined as the concurrent use of five or more medications [15,25]. This situation can lead to a heightened risk of adverse drug reactions, therapeutic duplication, and difficulties in medication adherence, exacerbating the challenges of managing such vulnerable populations [26,27,28]. Clinical physicians, before prescribing off-label drugs, need to evaluate the context of each patient’s clinical picture in an effort to find out possible pharmacological solutions and often to adapt a use from other contexts, but sometimes off-label prescribing is the only option available to ensure that the patient is provided with a therapeutic resource. In this sense, it is also necessary to work on improving access to known and reliable off-label uses to circumvent the long regulatory timeframe and to compensate families for covering the costs of these drugs, as they cannot always be included in their standard prescriptions regimen covered but the Italian National Health System [27].

Finally, in this sensitive process, a proper communication with patients and families about the risks and benefits associated with off-label use is mandatory. This complementary relationship should be implemented in clinical practice, fostering a comprehensive understanding of treatment options, facilitating informed and safe therapeutic decisions [17]. However, a significant limitation of our study is the lack of data on patients’ and families’ perspectives regarding off-label use. Understanding their views, concerns, and experiences is crucial for tailoring communication strategies and ensuring that therapeutic decisions align with their expectations and values. Future research should prioritize collecting and integrating these perspectives to provide a more holistic approach to off-label prescribing. By addressing this gap, we can enhance shared decision-making processes and strengthen trust between healthcare providers and patients, ultimately improving the overall quality of care.

As previously discussed, the use of off-label drugs in pediatrics raises many challenges for both pharmacists and pediatricians for the near future. While teamwork is essential for ensuring the safety and effectiveness of off-label medications in children, several barriers hinder its smooth implementation in research and clinical practice. Common obstacles include communication gaps, professional silos, resource constraints, and legal and ethical considerations. Limited understanding of off-label use among different professions (doctors, pharmacists, nurses) can lead to miscommunication and differing viewpoints. Busy schedules and inadequate channels for exchanging information between departments and institutions can further impede profitable collaboration. Moreover, territorial mindsets can emerge, where professionals hesitate to share knowledge or collaborate due to concerns about their autonomy or a perceived threat to their expertise. Additionally, differing priorities and goals across professional categories can create friction and hinder collaborative efforts. Time and funding shortages can restrict the resources available for multidisciplinary research, limiting the time and personnel needed for collaboration. The lack of dedicated staff, such as pharmacists or specialists in drug interactions, can slow down the implementation of effective medication management strategies for children. Furthermore, inadequate infrastructure, like electronic health record systems that do not support information sharing across disciplines, can also impede collaboration.

Finally, the complex legal and regulatory frameworks surrounding off-label medication use can create uncertainty and discourage collaboration. Ethical considerations regarding data sharing, patient privacy, and informed consent can also pose challenges in multidisciplinary research efforts [29]. Healthcare providers have to ensure the collection of informed consent, particularly in cases where evidence is limited or safety profiles are not fully established, making transparent communication with families about potential risks and benefits crucial to upholding trust and autonomy.

Ultimately, from an economic point of view, off-label prescribing can impact healthcare resources, requiring careful evaluation of cost-effectiveness and the financial burden on families [30]. By acknowledging these challenges and fostering a more collaborative environment, supported by multidisciplinary efforts and evidence-based guidelines, we can ensure better healthcare outcomes for children requiring off-label medications.

Collaboration between clinicians and pharmacists has been shown to significantly reduce the risk of medication errors related to the use of off-label drugs, but this is not systematic and is diffused in many clinical contexts [31,32]. The first role of pharmacists is to provide critical insights regarding appropriate doses, routes of administration, potential drug interactions, and the monitoring of side effects [25]. But to date there are few registries for pediatric off-label drugs or ongoing clinical trials dedicated to monitoring the use of these drugs and potentially giving more insights on the use, dosage, route of administration, safety, and effectiveness. Another important resource is the preparation of customized galenic formulations to meet the specific needs of the pediatric patient and provide correct information and training to facilitate drug delivery and solve some of these off-label indications [32]. The pharmacist can also play an important role in training healthcare personnel and informing patients and their families on the correct use of medicines [32,33,34]. Continuing education and regular updates on regulations are essential for all healthcare providers involved in the use of off-label drugs in pediatrics. These programs should focus on fostering a shared understanding between physicians and pharmacists, ensuring both groups are well-informed about regulatory frameworks, evidence-based practices, and the nuances of off-label prescribing. An interdisciplinary approach is critical to enhancing patient safety and ensuring optimal treatment outcomes [35]. Regularly scheduled multidisciplinary meetings, collaborative audits, and joint training sessions can create a platform for open dialogue, where healthcare providers can address uncertainties, align their perspectives, and refine treatment plans together. This collaboration is particularly important in managing complex cases involving polypharmacy or fragile pediatric patients, where decisions require a balanced integration of clinical judgment and pharmacological expertise [36]. By strengthening interdisciplinary communication and leveraging the unique contributions of each professional, healthcare teams can ensure that off-label prescribing aligns with both clinical needs and regulatory standards. This unified approach not only promotes safer and more effective care but also fosters a culture of shared responsibility and continuous improvement in pediatric pharmacotherapy [24,25].

Despite the valuable insights gained from our analysis, this study has several limitations. First, the sample size was relatively small, which may affect the generalizability of the findings. A larger and more diverse population, possibly obtained from a national, or even international, multicenter study, could provide a more comprehensive understanding of the complexities surrounding off-label drug use. Additionally, the reliance on self-reported data from physicians and pharmacists may have introduced bias, as responses could have been influenced by personal experiences or perceptions. Lastly, the study did not account for long-term outcomes associated with off-label use, which warrants further investigation.

Regarding potential divergences of interpretation between healthcare professionals, a deeper exploration of the root causes behind discrepancies in perspectives between pharmacists and physicians, including the impact of communication gaps and differences in training or clinical priorities, is essential to promote collaborative decision-making and optimize off-label prescribing practices.

To generate scientific evidence and improve patient safety related to the use of off-label drugs in pediatrics, the use of registries and databases should be implemented to promote participation in pediatric clinical trials.

**Figure 4:**
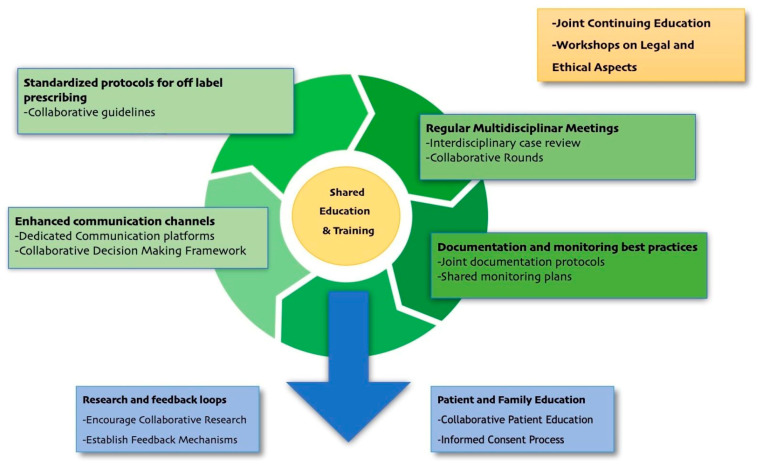
Call-to-action interventions proposed.

Looking ahead, further clinical research is essential to establish clear guidelines for off-label drug use in PPC, ideally through specific funding and the simplification of regulatory procedures. The creation of national and international registries to collect data on off-label drug use (especially for rare conditions) can provide valuable information for risk–benefit assessment and clinical guideline development as well as collaboration between healthcare institutions, universities, and pharmaceutical industries. See Figure 4 for a summary of the proposed initiatives.

## 5. Conclusions

Although a clear and flexible regulatory framework is already present in Italian law, there is still a clear need for regulatory authorities to implement streamlined evaluation mechanisms that facilitate off-label drug use in emergency situations or when there is an unmet clinical need [37]. This could involve expedited procedures for temporary approvals based on preliminary yet promising evidence. A more flexible regulatory framework, alongside potential developments in customized galenic formulations, could provide additional support in addressing the challenges of off-label drug use in pediatric palliative care. Moreover, a collaborative approach involving both physicians and pharmacists is essential for accurately identifying scenarios where off-label use is justified. This necessitates a thorough evaluation of the available evidence, expected benefits, and associated risks.

In conclusion, fostering increased collaboration between healthcare providers, alongside the adoption of adaptable regulatory measures, will ensure the safe and effective use of off-label medications, ultimately benefiting pediatric patients. This integration might range from investigating the effects of regular medication reviews, which could reveal significant insights for optimizing drug therapy, to supporting and delivering effective patient education on drug adherence to empower patients and their caregivers, and ultimately improving care for vulnerable patients.

## Figures and Tables

**Table 1:** Most common off-label categories.

Category	Description
Age	The medication is used in a population that is younger or older than the approved age range.
Indication	The drug is prescribed for a condition or disease that is not included in the approved indications.
Dosage	The dosage prescribed differs from the recommended dosage in the approved labeling.
Route of administration	The method of administration (e.g., oral, intravenous) is not specified in the SmPC.
Formulation	The medication is used in a different formulation than that approved (e.g., liquid instead of tablet).
Patient Population	Use in specific subpopulations not studied or approved, such as those with renal or hepatic impairment or children.

**Table 2:** Baseline characteristics of the cohort. SD: standard deviation; IQR: interquartile range.

	n (%)	Mean (SD)	Median (IQR)
Total population	169		
Male	83 (49%)		
Female	86 (51%)		
Age (years)		11.2 (±5.9)	12.5 (6–18)
Infants (1 month–2 years)	10 (5.9%)		
Preschool (3–5 years)	33 (19.5%)		
School (6–11 years)	38 (22.5%)		
Adolescent (12–18 years)	65 (38.5%)		
Adult (≥18 years)	23 (13.6%)		
Disease history (years)		10.4 (±6.0)	11 (2–18)
Disease—details			
Cardiac	2 (1.2%)		
Musculoskeletal	48 (28.4%)		
Neurologic	70 (41.4%)		
Oncological	11 (6.5%)		
Respiratory	11 (6.5%)		
Genetic–metabolic	27 (16.0%)		
Congenital/Perinatal onset	153 (90.5%)		
Drug Burden			
Drugs taken per day		5.9 (±3.1)	
Patients experiencing polypharmacy (≥5 drugs per day)	89 (52.7%)		
Patients experiencing a medication burden	75 (44.4%)		
Self-administration	38 (22.5%)		
Patients experiencing excessive polypharmacy (≥10 drugs per day)	33 (19.5%)		
Number of drugs taken per day in excessive polypharmacy patients		13.5 (±2.1)	
Medication Regimen Complexity Index (MRCI) for excessive polypharmacy patients		44.8 (±12.5)	

**Table 3:** Off-label categories analysis divided between clinicians and pharmacists.

	Physicians Group—n (%)	Hospital Pharmacists Group—n (%)	*p*-Value
Analyzed prescriptions	951 (100)	951 (100)	
Identified off-label uses	175 (18.4)	313 (32.9)	**<0.05**
Off-label classification: use for different indication	100 (57.1)	81 (25.9)	**<0.05**
Off-label classification: use not authorized in age group	68 (38.8)	106 (33.9)	0.27
Off-label classification: not recommended route of administration	8 (4.6)	31 (9.9)	0.04
Off-label classification: not recommended dosage	32 (18.3)	138 (44.1)	**<0.05**
Off-label classification: other not recommended uses (e.g., unauthorized manipulation)	30 (17.1)	3 (0.9)	**<0.05**

## Data Availability

Data are available upon request to the corresponding authors due to privacy reasons.

## Abbreviations

The following abbreviations are used in this manuscript:

PPC	Pediatric Palliative Care
ADE	Adverse Drug Event
SmPC	Summary of Product Characteristics
AIFA	Agenzia Italiana del Farmaco—Italian Medicines Agency
CDSS	Clinical Decision Support Software
ATC	Anatomical Therapeutic Chemical
GCP	Good Clinical Practice
EC	Ethical Committee
